# The Role of Pulse Shape in Motor Cortex Transcranial Magnetic Stimulation Using Full-Sine Stimuli

**DOI:** 10.1371/journal.pone.0115247

**Published:** 2014-12-16

**Authors:** Igor Delvendahl, Norbert Gattinger, Thomas Berger, Bernhard Gleich, Hartwig R. Siebner, Volker Mall

**Affiliations:** 1 Carl-Ludwig-Institute for Physiology, Leipzig University, Leipzig, Germany; 2 Zentralinstitut für Medizintechnik, Technische Universität München (IMETUM), Garching, Germany; 3 Department of Pediatrics and Adolescent Medicine, University Medical Center Freiburg, Freiburg, Germany; 4 Danish Research Center for Magnetic Resonance, Copenhagen University Hospital Hvidovre, Copenhagen, Denmark; 5 Department of Neurology, Copenhagen University Hospital Bispebjerg, Copenhagen, Denmark; 6 Department of Pediatrics, Technische Universität München, Munich, Germany; IIT - Italian Institute of Technology, Italy

## Abstract

A full-sine (biphasic) pulse waveform is most commonly used for repetitive transcranial magnetic stimulation (TMS), but little is known about how variations in duration or amplitude of distinct pulse segments influence the effectiveness of a single TMS pulse to elicit a corticomotor response. Using a novel TMS device, we systematically varied the configuration of full-sine pulses to assess the impact of configuration changes on resting motor threshold (RMT) as measure of stimulation effectiveness with single-pulse TMS of the non-dominant motor hand area (M1). In young healthy volunteers, we (i) compared monophasic, half-sine, and full-sine pulses, (ii) applied two-segment pulses consisting of two identical half-sines, and (iii) manipulated amplitude, duration, and current direction of the first or second full-sine pulse half-segments. RMT was significantly higher using half-sine or monophasic pulses compared with full-sine. Pulses combining two half-sines of identical polarity and duration were also characterized by higher RMT than full-sine stimuli resulting. For full-sine stimuli, decreasing the amplitude of the half-segment inducing posterior-anterior oriented current in M1 resulted in considerably higher RMT, whereas varying the amplitude of the half-segment inducing anterior-posterior current had a smaller effect. These findings provide direct experimental evidence that the pulse segment inducing a posterior-anterior directed current in M1 contributes most to corticospinal pathway excitation. Preferential excitation of neuronal target cells in the posterior-anterior segment or targeting of different neuronal structures by the two half-segments can explain this result. Thus, our findings help understanding the mechanisms of neural stimulation by full-sine TMS.

## Introduction

Transcranial magnetic stimulation (TMS) is commonly used to non-invasively probe and alter human motor cortex excitability via a time-varying magnetic field [1]. In principle, a magnetic stimulator consists of a capacitor that is linked via a power-switch to a stimulation coil consisting of, for instance, multiple wound copper wires. This design leads to a serial resonant circuit with a sinusoidal high-frequency current flow through the stimulation coil as long as the power-switch is closed. Many TMS devices deliver a so-called monophasic pulse, where the pulse current is dampened after the first quarter cycle resulting in a monodirectional (i.e. of single polarity) current flow. Monophasic pulses are particularly suited to study direction specific stimulation effects [2]. Because of the low energy efficacy, a monophasic waveform is mainly used to probe cortical excitability with single or paired pulses [3].

When stopping the current flow after the second quarter cycle, a half-sine pulse results. A third stimulus type, the so-called biphasic waveform, is achieved if current is allowed to flow for a full-sine cycle. In contrast to monophasic and half-sine pulses, full-sine stimuli are composed of two half-segments with opposite current direction. A main advantage of full-sine (biphasic) stimulators is energy efficacy because much of the energy can be re-used. Stimulators outputting a full-sine waveform allow repetition rates of more than 50 pulses per second, and are hence primarily employed for repetitive stimulation [4], [5]. Repetitive stimulation paradigms are very useful to induce plasticity in the human brain [6], to temporarily disrupt function of specific brain areas [7], and to treat brain disorders [8], [9]. Importantly, the stimulation effect with all three TMS waveforms depends strongly on the direction of current induced in the tissue. TMS pulses inducing a posterior-anterior (PA) current flow are associated with lower threshold intensity to induce a motor-evoked potential (MEP) and shorter MEP latency as opposed to TMS pulses inducing anterior-posterior (AP) current in the primary motor hand area (M1-HAND). The strong direction-dependent effect of TMS can most likely be attributed to activation of different neuronal structures by PA and AP oriented pulses [10].

Recently, new stimulation devices have been introduced that allow modulation of pre-existing pulse waveforms and application of novel TMS waveforms [11], [12]. Furthermore, recent studies highlighted the physiological importance of TMS waveform parameters, showing that pulse waveform and current direction have a strong influence on single- and paired-pulse measurements of cortical excitability [13]–[16] as well as on plasticity-inducing repetitive TMS protocols in human M1-HAND [17]–[21]. This body of research motivated the present study to assess the physiological properties of novel TMS waveforms and to investigate how modulations of a given waveform influence cortex stimulation.

Novel stimulation devices provide the opportunity to empirically test the impact of TMS waveform modulations on the ability to evoke an MEP. Such in vivo tests are critical because many stimulation effects cannot readily be simulated, such as the strong dependence on current direction. In this context, the full-sine waveform is of great interest as it is most frequently used in studies applying repetitive TMS (rTMS) to induce plasticity or to interfere with a given brain function. Several studies indicated that cortical neurons are more sensitive to full-sine than to monophasic or half-sine stimulation. With full-sine pulses, a lower stimulation threshold for inducing MEPs over M1-HAND or phosphenes over visual cortex, respectively, was observed [13], [22]–[25], indicating higher effectiveness. Here, “effectiveness” is defined in terms of “pathway excitation” rather than spatial focality or capability to induce lasting changes in intrinsic excitability. It was postulated that the second and third quarter cycle of the full-sine waveform are physiologically relevant for the stimulation effect, because the influence of current direction is reversed for full-sine pulses relative to monophasic or half-sine [13], [26], [27]. However, it remains unclear whether the duration or amplitude of a given pulse segment renders the full-sine waveform more effective and how an alteration of pulse segments impacts on cortical stimulation. The more flexible pulse design of recently developed stimulation devices [12] thus offers the chance to advance our understanding of the stimulation effect for the full-sine waveform.

In this study, we directly examine which waveform characteristics are responsible for the stimulation effect of full-sine TMS in human M1-HAND. Using several novel TMS pulse shapes we investigate how alterations of a given half-segment of the full-sine pulse waveform impact on motor threshold. Our findings elucidate how duration, amplitude, order, and polarity of the two half-sine coil current segments influence excitation of corticospinal neurons in human motor cortex by full-sine stimuli.

## Materials and Methods

### Participants

**Figure 1:**
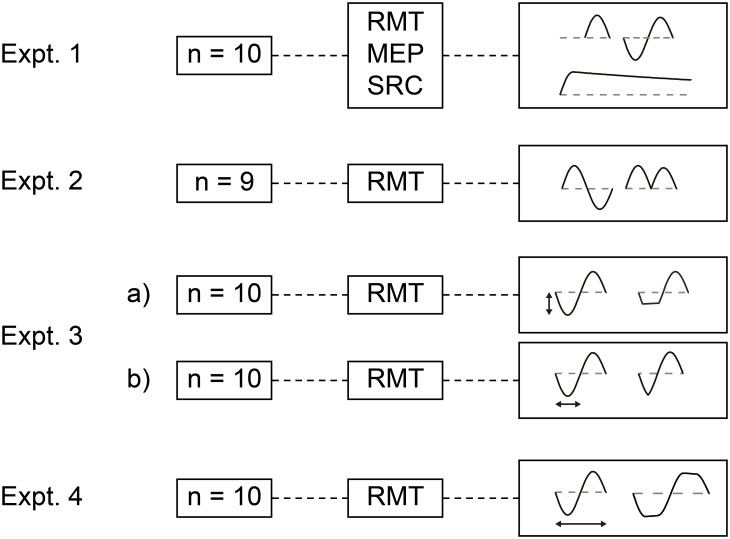
Experimental design. The study consisted of four experiments. The flexTMS stimulator allowed a comparison of the three conventional TMS waveforms applied by a single stimulation device (Expt. 1). We probed resting motor threshold (RMT), MEP amplitude, and stimulus-response-curves (SRC). In experiment 2, full-sine TMS pulses were compared with waveforms consisting of two concatenated half-sine pulses of identical current orientation. Expt. 3 examined how modifications of a given half-segment of the full-sine pulse impact on RMT. Changes in amplitude (Expt. 3a) and duration (Expt. 3b) of a given half-segment were tested separately. The duration of full-sine pulses was extended covering a longer range in experiment 4. See *Methods* for a detailed description of experimental procedures.

The local Ethics Committees of the University Medical Center of Freiburg, Germany approved the study (approval number 425/11), which was carried out according to the latest version of the Declaration of Helsinki. Twenty-four healthy volunteers aged 25.5±0.7 years (12 women, 12 men) participated after having given written informed consent. None of the participants had a history of neurological illnesses, took any CNS-active medication at the time of testing, or met one of the exclusion criteria published in the safety guidelines for TMS [28]. Twenty-two participants were right-handers and two participants were left-handers according to self-report. The study consisted of four experiments (Fig. 1) in partly separate groups of participants; experimental sessions were separated by at least one week to avoid possible carry-over effects.

### Electromyographic recording

Participants were seated comfortably in an armchair with their stimulated hand resting on a cushion. MEPs were recorded by surface electromyography from the fully relaxed abductor pollicis brevis muscle of the non-dominant hand using silver/silver chloride electrodes (surface area, 263 mm^2^; AMBU, Ballerup, Denmark) and a bipolar belly-tendon montage. Data were band-pass filtered (20–2000 Hz), amplified (Ekida DC universal amplifier, EKIDA GmbH, Helmstadt, Germany), digitized at a sampling rate of 5 kHz (MICRO1401mkII data acquisition unit, Cambridge Electronic Design Ltd, Cambridge, UK), and stored on a personal computer for online visual display and later offline analysis using Signal Software version 3 (Cambridge Electronic Design Ltd, Cambridge, UK). Participants were asked to relax the target muscle throughout the experiment, and muscle relaxation was monitored using online visual feedback of electromyographic activity in the target muscle.

### Transcranial magnetic stimulation

We performed TMS with a flexTMS stimulator (IMETUM, Garching, Germany). The technical details of this stimulation device have previously been described in detail [12]. The intrinsic resonance frequency of the flexTMS device results in an unaltered full-sine waveform of 160 µs duration, and half-sine pulses with 80 µs duration, correspondingly. Based on these standard waveforms we designed the pulses for this study using custom-made software [12]. Different types of coil current waveforms were created consisting of up-, down-, and hold-segments. The concatenation of these three segment types enables flexible design and application of pulse waveforms within certain technical limits [12]. Stimulus waveform templates generated by software were then transferred to the stimulation device.

**Figure 2:**
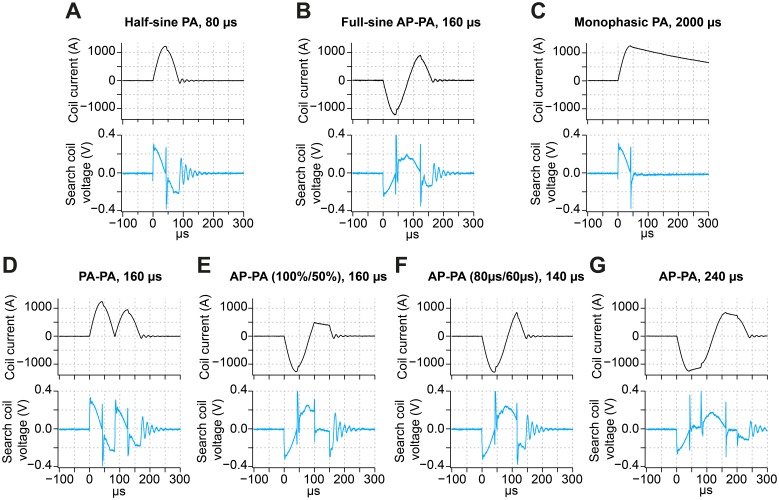
Waveforms applied by the flexTMS device. Depicted are recorded waveforms used in the four experiments (see *Methods*). Traces show coil current waveform (black traces) and the induced voltage of the search coil (light blue traces). The search coil output voltage is proportional to the induced electric field. All pulse shapes were measured at 25% MSO. (A, B, C) Shown are recorded pulses for the half-sine, full-sine, and monophasic waveforms used in Expt. 1. These waveforms are commonly used in TMS. (D) Depicted is the two segment waveform consisting of two concatenated PA-oriented half-sines used in Expt. 2. (E) In Expt. 3a, the amplitude of a given half-segment of the full-sine pulse was modified. In the shown pulse waveform, the second half-segment was reduced to 50% of the unaltered AP-PA pulse. (F) In Expt. 3b, the duration of a given half-segment of the full-sine pulse was modified. In the illustrated pulse waveform, the second half-segment of the AP-PA pulse was shortened to 60 µs. (G) The symmetrically prolonged full-sine pulses probed in Expt. 4 induce an electric field waveform where the opposite phases are temporally separated.

We recorded coil current and electric field waveforms essentially as previously described [12]. Pulse shapes were applied with a figure-eight air core coil (P/N 510519, MAG & More GmbH, Munich, Germany). The coil current and electric field were measured with a Rogowski current probe (CWT60B, Power Electronics Measurements Ltd, Nottingham, UK) and a search coil, respectively. The search coil, made of a five-turn circular winding with outer diameter of 2 cm and inner diameter of 0.5 cm, was positioned parallel to the TMS coil plane with a distance of approximately one millimeter at the focus of the figure-eight coil. The search coil output voltage is proportional to the electric field [29]. Data were sampled at 200 MHz and digitally filtered to 5 MHz (−3 dB cutoff) using the finite impulse response filter tool in Igor Pro software (Wavemetrics, Lake Oswego, OR, USA). Coil currents and induced voltages of waveforms used in the experimental sessions are presented in Fig. 2. In the remaining figures, waveform pictograms are simplified for clarity.

### Transcranial magnetic stimulation

TMS targeted the non-dominant M1-HAND. Single pulses were applied trough a figure-eight shaped stimulation coil with an outer diameter of 100 mm (MAG & More GmbH, Munich, Germany) connected to the flexTMS device. TMS was triggered manually and administered to M1-HAND at inter-sweep intervals of 5–10 s. The stimulation coil was centered tangentially on the scalp over M1-HAND with its handle pointing in a posterior direction and laterally at an angle of approximately 45° away from the midline. At this coil orientation, TMS-induced current in M1-HAND was optimal for activating corticospinal neurons trans-synaptically via horizontal cortico-cortical connections [30]. Current direction was reversed, if necessary, from posterior-anterior (PA) to anterior-posterior (AP) by altering waveform polarity without changing coil position. Throughout the manuscript, current direction refers to induced current in M1-HAND. We located the optimal position for eliciting MEPs from the target muscle, defined as coil position over M1-HAND that produced reliable MEPs of >50 µV with lowest stimulation intensity. This coil position was recorded and maintained using a stereotactic, optically tracked navigation system as described [31].

### Cortical motor threshold

The cortical resting motor threshold (RMT) was determined by a probabilistic threshold-hunting method using Motor Threshold Assessment Tool software (MTAT 2.0) [32], [33]. The TMS intensity to use for each trial is specified by the program and adjusted trial-by-trial based upon information whether an MEP was elicited or not. We always used 16 stimuli starting at 45% of maximum stimulator output (MSO) to determine RMT. The presence of an MEP was operationally defined as compound muscle action potential with peak-to-peak amplitude >50 µV. RMT was expressed as percentage of MSO. Within the four experiments, RMT measurements for the various stimulus conditions were performed in pseudo-randomized order.

In TMS, RMT can be used as measure of excitation threshold of the neuronal target elements [34]. For a given stimulation device, RMT thus directly reflects the effectiveness of neural stimulation, with lower RMT indicating a higher probability of successful suprathreshold membrane depolarization of neurons in M1-HAND [35]. The RMT is used in many studies to individually adjust the intensity of single-pulse and repetitive TMS, but the effectiveness of a given pulse waveform in rTMS protocols to induce lasting changes in cortical excitability might diverge from the effectiveness determined by RMT [21]. Since RMT is a reliable and physiologically relevant indicator of effectiveness of a single TMS pulse, we focused on measurements of RMT in the present study.

### Experimental design

#### Experiment 1

We first compared the three commonly used TMS waveforms (full-sine, half-sine, and monophasic) in 10 participants (25.3±0.6 years, four female, six male). The flexTMS device allowed ruling out potential influences of stimulator design on waveform effects. We investigated if the previously reported differences between stimulus waveforms [13], [26] can be reproduced if full-sine, half-sine, and monophasic pulses are applied with the same stimulation device. For all three waveforms, we determined RMT, measured MEP amplitude, and obtained stimulus-response-curves to characterize the increase in MEP size with stimulus intensity. Stimulus-response-curves were acquired with threshold-adapted stimulus intensities, i.e. 90%, 100%, 110%, 120% and 130% of individual RMT. We recorded 10 MEPs per stimulus intensity in a pseudo-randomized order to avoid hysteresis effects [36].

#### Experiment 2

In this experiment, we explored if the higher effectiveness of full-sine pulses can be attributed to longer duration of coil current flow or the sinusoidal pulse waveform. In nine participants (26.3±1.7 years, four female, five male), we tested the effect of four two-segment pulses on RMT: The common two current orientations of full-sine pulses (AP-PA and PA-AP) and pulses with both half-segments having the same current orientation (i.e. PA-PA and AP-AP pulses). These measurements enabled us to separate the influence of absolute pulse duration from effects of the biphasic full-sine pulse waveform.

#### Experiment 3

The two opposing half-segments and current orientations within the full-sine pulse might contribute differentially to the stimulation effect. In 10 participants (24.3±0.5 years, six female, four male) we varied amplitude (Expt. 3a) and duration (Expt. 3b) of a specific segment of the full-sine pulse waveform and tested the impact on RMT. For full-sine (biphasic) waveforms, the second half-segment is reduced by ∼10–20% due to damping effects. In this experiment, pulse amplitude in percent was therefore referenced to the unaltered 160 µs full-sine pulse. In experiment 3a, pulse segment amplitude was reduced from 100% to 75, 50, and 25%. In experiment 3b, the duration of either the first or the second segment of the pulse was changed from 80 µs to 60 or 100 µs, respectively. We performed these procedures separately for the two half-segments of the pulse and current orientations (i.e. AP-PA and PA-AP), and recorded RMT for each condition.

#### Experiment 4

The modified full-sine waveforms in the previous experiments changed the middle part of the induced voltage and hence the respective phase of the electric field (Fig. 2). To investigate waveform modifications that do not alter the middle part of the induced electric field waveform, we measured RMT with longer pulses derived from the full-sine waveform. The prolongation part of these pulses does not contribute much to the induced electric field and leaves the middle phase of the waveform unaltered. Ten participants (26.6±1.6 years, four female, six male) took part in this experiment. Biphasic pulses of 200 and 240 µs duration were compared with the unchanged 160 µs full-sine pulse.

### Data analysis

MEP peak-to-peak amplitude was determined with Signal software and averaged over 10 trials. Statistical analysis was done using SPSS version 18.0 Software (SPSS Inc., Chicago, IL, USA). Normality of data was confirmed using the Kolmogorov-Smirnov test. Statistical evaluation was performed via repeated-measures analysis of variance (rmANOVA). To compare the three principal waveforms (Expt. 1), rmANOVAs with the factor waveform (3 levels: monophasic, half-sine, and full-sine) were performed with RMT, MEP amplitude, and MEP latency as dependent variables. In Experiment 2, the within-subjects factor waveform (4 levels: AP-PA, PA-AP, PA-PA, and AP-AP) was entered for rmANOVA with RMT as dependent variable. For experiment 3, where the two segments of the full-sine waveform were varied separately, we used a three-way rmANOVA. For statistical analysis of this experiment, RMT data were normalized to the unaltered standard full-sine pulse. In Expt. 3a, pulse amplitude (3 levels: 75%, 50%, and 25%), current direction (2 levels: AP/PA and PA/AP) and pulse segment (2 levels: first and second half-segment) were used as within-subjects factors, and pulse length (2 levels: 140 µs and 180 µs), current direction (2 levels: AP/PA and PA/AP) and pulse segment (2 levels: first and second half-segment) in Experiment 3b. For Experiment 4, rmANOVA included the within-subjects factor duration (3 levels: 160 µs, 200 µs, and 240 µs). We used the Greenhouse-Geisser correction to adjust for violations of sphericity, if necessary. In case of significant interaction or main effects, rmANOVA was followed by separate two-way rmANOVAs, where applicable, and by post-hoc analysis using two-tailed Student’s paired or one-sample *t* tests. Multiple comparisons were corrected using the Bonferroni-Holm method. Level of statistical significance was set at *P*<0.05. Data are expressed as means ± sem except where stated.

## Results

All participants tolerated stimulation with the flexTMS device well without reporting any adverse effects or spread of excitation to neighboring hand or forearm muscles. Mean RMT of all participants determined with full-sine AP-PA pulses of 160 µs duration was 59.2±2.0% MSO.

**Figure 3:**
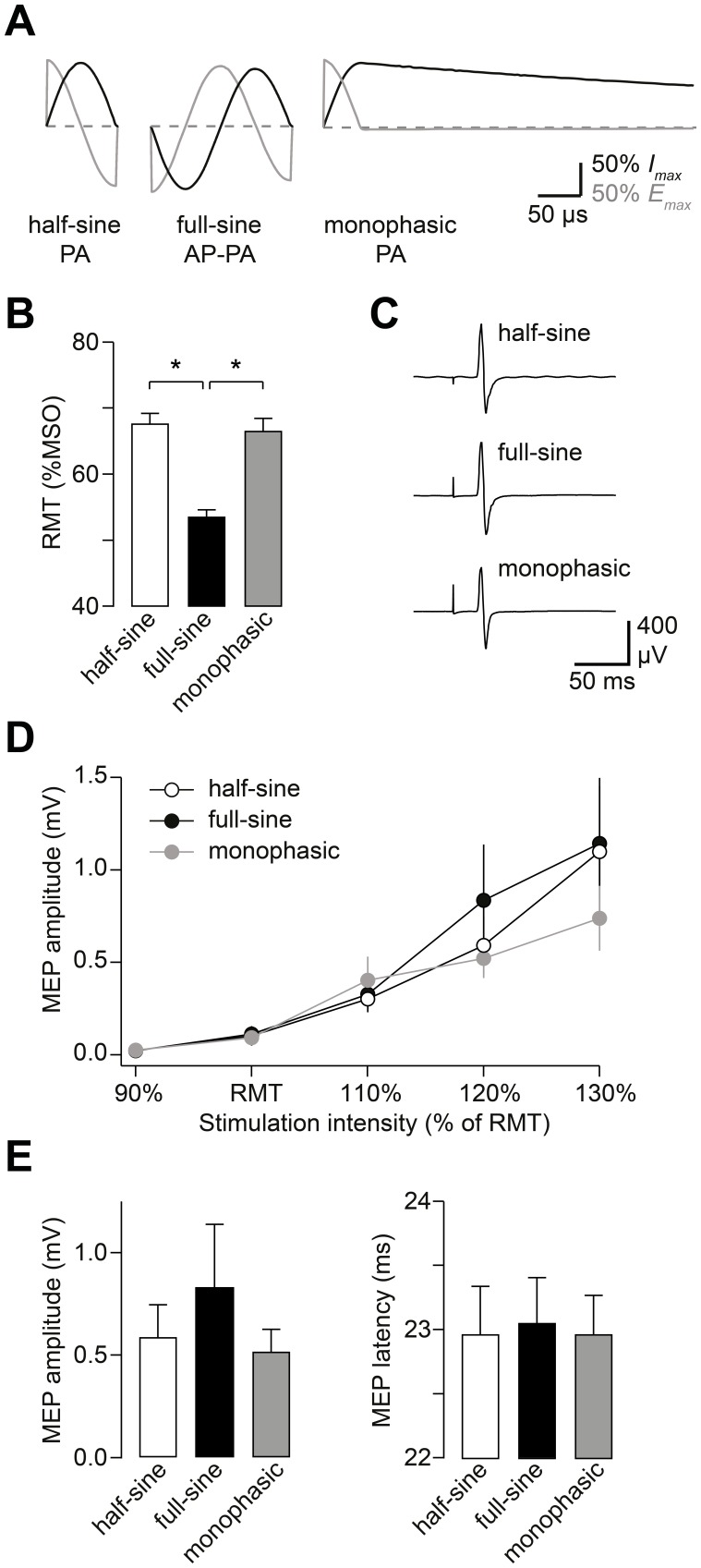
Comparison of the three different TMS waveforms using a single stimulation device. (A) The three commonly used TMS waveforms. The flexTMS device allows application of half-sine, full-sine and monophasic pulses. Bold lines indicate the coil current *I*, gray lines represent the induced electric field *E*. The monophasic pulse is truncated at 400 µs for display purposes. All waveform pictograms in this and the following figures are simplified for illustration (see Fig. 2 for recorded waveform traces). (B) Mean RMT for the three different waveforms applied with the same stimulator. RMT was significantly lower when probed with full-sine pulses, indicating higher effectiveness of this waveform to elicit a motor response. (C) MEPs evoked with half-sine, full-sine, and monophasic stimuli from a representative participant. Traces are averages of 10 trials each and were recorded using stimulation intensity (SI) of 120% of RMT. (D) Stimulus-response curves for half-sine, full-sine (biphasic), and monophasic stimuli. Depicted is the mean MEP amplitude as a function of SI for each waveform. Stimulus-response curves were recorded at intensities referenced relative to individual RMT. (E) Mean amplitude and latency of MEPs recorded using SI of 120% of RMT for the three different waveforms. Data are from n = 10 participants, error bars represent sem. *P<0.05, Student’s paired *t* test.

To rule out that differences in stimulator design contribute largely to the different effectiveness of TMS waveforms [37], we stimulated M1-HAND probing the three conventional waveforms with the flexTMS device (Experiment 1, Fig. 3A). As expected, half-sine, full-sine, and monophasic pulse waveforms differed with respect to RMT (rmANOVA factor waveform: *F*_(2;18)_ = 61.07, *P*<0.0001; Fig. 3B). Post-hoc testing revealed that RMT was significantly lower with full-sine TMS pulses than with half-sine (*P*<0.0001, Student’s paired *t* test) or monophasic pulses (*P*<0.0001, Student’s paired *t* test).

We next investigated the increase in MEP amplitude with increasing stimulus intensity for the three TMS waveforms. Fig. 3C shows representative MEP traces elicited by half-sine, full-sine and monophasic waveforms in one participant. Stimulus-response curves recorded at intensities relative to individual RMT were comparable for all three waveforms (Fig. 3D). In rmANOVA, there was a consistent main effect of intensity (*F*_(1.2;11.2)_ = 11.73, *P* = 0.004), but no effect of waveform (*F*_(2;18)_ = 0.22, *P* = 0.21), and no interaction between intensity and waveform (*F*_(1.6;14.5)_ = 0.94, *P* = 0.40). We also determined the slope of individual stimulus-response-curves with linear regression of the data points recorded at suprathreshold intensity. Slopes did not differ among the three waveforms (rmANOVA: *F*_(2;18)_ = 2.09, *P* = 0.15). The finding that slope and shape of stimulus-response-curves did not differ between half-sine, full-sine, and monophasic waveforms indicates that although RMT is lower using full-sine stimulation, all three pulse waveforms induce a comparable gain in corticospinal excitation with increasing stimulus intensity [38]. However, the limited range of stimulus intensities allowed only probing the initial rising flank of the stimulus intensity curve, which does not exclude waveform dependent differences in MEP amplitudes at higher stimulus intensities.

For single-pulse MEPs recorded at 120% of RMT with each of the three different waveforms (Fig. 3E), there was no effect of waveform on MEP amplitude (rmANOVA: *F*_(2;18)_ = 0.91, *P* = 0.42) or MEP latency (rmANOVA: *F*_(2;18)_ = 0.15, *P* = 0.86). The comparable latency supports the assumption that PA oriented half-sine and monophasic as well as AP-PA oriented full-sine pulses recruit similar descending waves in the corticospinal tract [14], [39].

**Figure 4:**
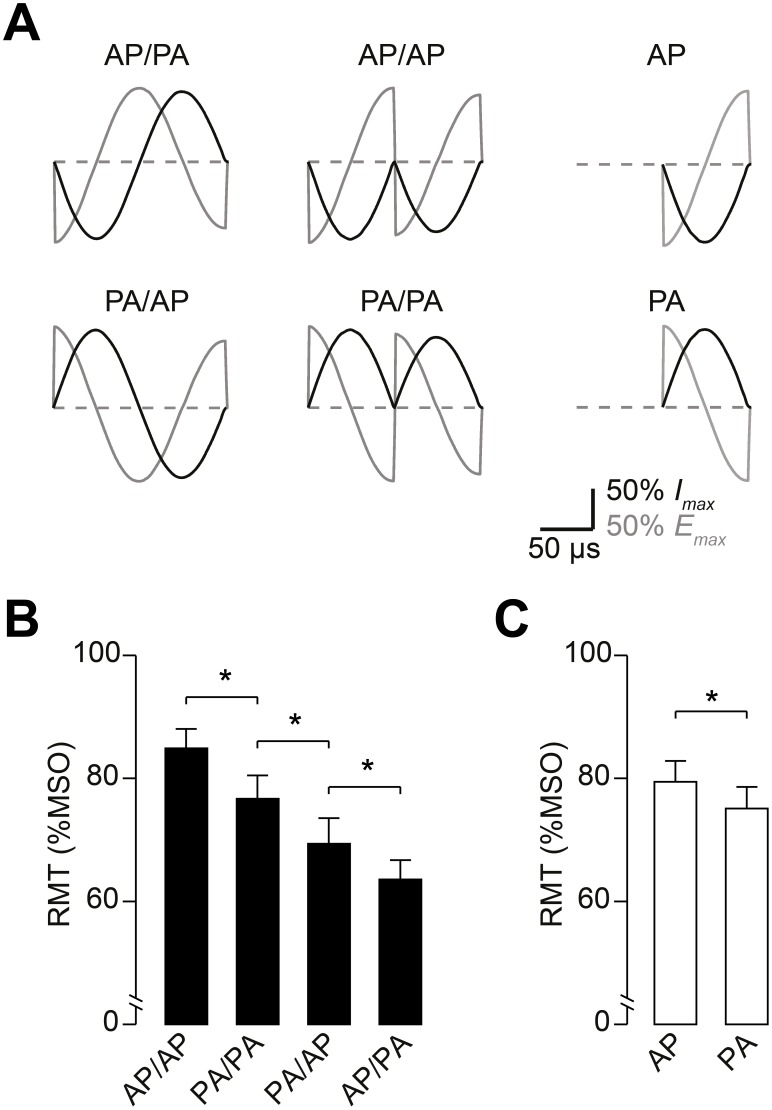
Full-sine waveforms have lower RMT than pulses with two half-sine segments of identical current orientation. (A) TMS pulse waveforms used for this experiment. Pulses of 160 µs duration were designed with two identical half-segments (termed AP/AP and PA/PA) to investigate the influence of pulse duration. (B) Depicted is mean RMT (n = 9) for full-sine stimuli (AP/PA and PA/AP) and concatenated stimuli with two identical half-sines (PA/PA and AP/AP). (C) For comparison, mean RMT is displayed for 80 µs half-sine stimuli (n = 10). Note that RMT was not lower for pulses with two concatenated half-sine half-segments (PA/PA and AP/AP; duration 160 µs) compared with the respective single half-sine pulses (duration 80 µs). Data are means, error bars represent sem. *P<0.05, Student’s paired *t* test.

Full-sine pulses have longer pulse duration than half-sine, which might contribute to the observed difference in RMT. In experiment 2, we investigated pulses of 160 µs duration that consisted of two half-segments with identical current orientation, referred to as PA-PA and AP-AP, respectively. We applied these two-segment pulses and the standard full-sine waveform using two initial current orientations, resulting in four stimulation conditions (AP-PA, PA-AP, PA-PA, and AP-AP; Fig. 4A). RMT was significantly different for these two-segment pulses (rmANOVA: main effect of waveform, *F*_(3;24)_ = 69.39, *P*<0.0001). There is a known dependence of RMT on current direction [24], [40], which was confirmed for all four two-segment pulses in this experiment. PA-AP and AP-AP oriented pulses had significantly higher RMT values compared with AP-PA and PA-PA, respectively (PA-AP vs. AP-PA: *P* = 0.03; AP-AP vs. PA-PA: *P*<0.001, Student’s paired *t* test, Fig. 4B). Likewise, a direction specific effect was observed for half-sine pulses; RMT was significantly higher for AP oriented stimulation than for PA current orientation (*P* = 0.003, paired *t* test; Fig. 4C). The absolute difference in RMT between the two current orientations was comparable for full-sine and half-sine stimulation (full-sine: 5.5±2.1% MSO (n = 9); half-sine: 6.8±1.7% MSO (n = 10); *P* = 0.64, unpaired *t* test). For the two-segment waveforms, pulses with two identically oriented segments had significantly higher RMT than full-sine waveforms (PA-PA vs. AP-PA: *P*<0.0001; AP-AP vs. PA-AP: *P*<0.0001, Student’s paired *t* test). In addition, the two-segment pulses with two identical half-segments yielded RMT values comparable to 80 µs half-sine pulses (PA-PA vs. PA: *P* = 0.75; AP-AP vs. AP: *P* = 0.55; unpaired *t* test). Thus, the lower RMT observed with full-sine pulses could not be reproduced by adding an identical half-segment to a half-sine pulse. These findings demonstrate that the shape of the waveform determines effectiveness of full-sine pulses. In contrast to half-sine or concatenated pulses, full-sine pulses have a long middle phase in the electric field waveform that lasts a half-cycle and has higher amplitude. This feature is most likely responsible for the lower RMT observed with full-sine pulses [26], [37].

**Figure 5:**
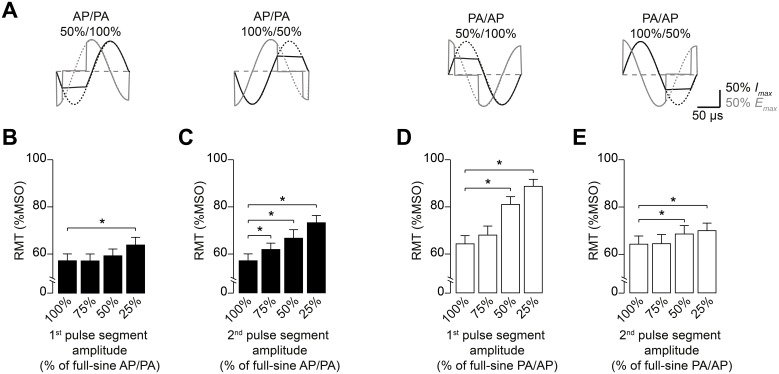
Changing half-segment amplitude of the full-sine pulse differentially influences RMT. To test if the half-segments inducing AP or PA current in M1-HAND differentially influences RMT, the amplitude of either the first or second half-segment of the full-sine pulse was altered for both current orientations separately. (A) Examples of waveforms used for this experiment. Coil current amplitude *I* was reduced to 75%, 50%, or 25% for a given half-segment. Pulse amplitudes in percent refer to amplitude of the first or second half-segment of the unaltered full-sine waveform (i.e. 100%/100% relative amplitude, illustrated by dashed lines). (B) Amplitude variation of the first half-segment of the AP/PA pulse resulted in minor changes of RMT. (C) Reduction of the second half-segment’s amplitude of the AP/PA pulse produced a significant increase in RMT already at 75% relative pulse segment amplitude. (D) Amplitude variation of the first segment of the PA/AP pulse led to a strong increase in RMT. (E) Amplitude variation of the second segment of the PA/AP pulse resulted in small changes of RMT. Data are means (n = 10), error bars represent sem. *P<0.05, one-sample *t* test on data normalized to the unaltered reference pulses.

The two half-segments of the full-sine pulse inducing opposite current flow in the tissue generate the long middle phase of the electric field waveform. However, the two half-segments might contribute differentially to the stimulation effect [13]. In addition, the direction of initial current flow influences stimulation with full-sine pulses [13], [24], [26], [41]. Therefore, we investigated the relative contribution of the opposing half-segments for the two current orientations within the full-sine pulse. To this end, we varied the amplitude of either the first or the second half-segment of the full-sine waveform (Experiment 3a, Fig. 5A) and probed AP-PA and PA-AP current orientations to capture possible influences of current direction. Because the current direction (PA or AP) of the second half-segment may explain the orientation specific results of the full-sine waveform (Fig. 4), we hypothesized that an amplitude reduction in the second half-segment has stronger influence on RMT than alterations in the first half-segment. RmANOVA with normalized RMT as dependent variable revealed a significant amplitude×current direction×pulse segment interaction (*F*_(1.3;11.2)_ = 27.1, *P*<0.001; see Table 1 for details). Separate follow-up rmANOVAs for both pulse segments yielded a significant amplitude×current direction interaction (first half-segment: *F*_(1.3;11.9)_ = 19.8, *P*<0.001; second half-segment: *F*_(2;18)_ = 6.1, *P* = 0.01), demonstrating a differential effect of amplitude changes for the AP and PA oriented segments of the full-sine pulse. A reduction of pulse amplitude had a significantly greater impact if the waveform was modulated in the PA oriented half-segment, irrespective of whether this was the first or second segment (Fig. 5C and 5D; Table 2). Thus, the relative contribution of a given half-segment depends on current orientation, which is determined by the order of the two opposite half-segments within the pulse.

**Table 1:** Results of three-way rmANOVAs conducted for experiment 3.

Waveform modulation	Factor	df	*error*	*F = *	*P = *
Amplitude (Expt. 3a)	amplitude	1.19	10.68	121.29	**<0.0001**
	pulse segment	1	9	5.74	**0.04**
	current direction	1	9	5.12	0.05
	pulse segment×current direction	1	9	91.53	**<0.0001**
	pulse segment×amplitude	2	18	5.67	**0.012**
	current direction×amplitude	2	18	4.24	**0.031**
	pulse segment×current direction×amplitude	1.25	11.24	27.10	**<0.001**
Pulse length (Expt. 3b)	pulse length	1	9	34.95	**<0.001**
	pulse segment	1	9	19.45	**0.002**
	current direction	1	9	0.32	0.59
	pulse segment×current direction	1	9	4.39	0.066
	pulse segment×pulse length	1	9	0.09	0.77
	current direction×pulse length	1	9	7.85	**0.021**
	pulse segment×current direction×pulse length	1	9	0.04	0.85

**Table 2:** The influence of changing the amplitude or duration of one segment of the full-sine waveform depends on current orientation.

Experiment 3a				
Relative amplitude(a)		Normalized RMT(b)		
First segment	Second segment	AP-PA(c)	PA-AP(c)	*P* = (d)
75%	100%	1.00±0.02	1.06±0.03	**0.022**
50%	100%	1.04±0.02	1.27±0.02	**<0.00001**
25%	100%	1.12±0.02	1.39±0.04	**0.0003**
100%	75%	1.09±0.02	1.00±0.02	**0.0019**
100%	50%	1.17±0.03	1.07±0.02	**0.0049**
100%	25%	1.29±0.03	1.09±0.02	**0.0002**

Experiment 3b				
Absolute duration(e)		Normalized RMT(b)		
First segment	Second segment	AP-PA(c)	PA-AP(c)	*P* = (d)
60 µs	80 µs	1.00±0.03	0.99±0.02	0.79
100 µs	80 µs	0.92±0.02	0.95±0.02	0.14
80 µs	60 µs	1.06±0.03	1.02±0.03	**0.04**
80 µs	100 µs	0.98±0.03	0.97±0.02	0.60

**Figure 6:**
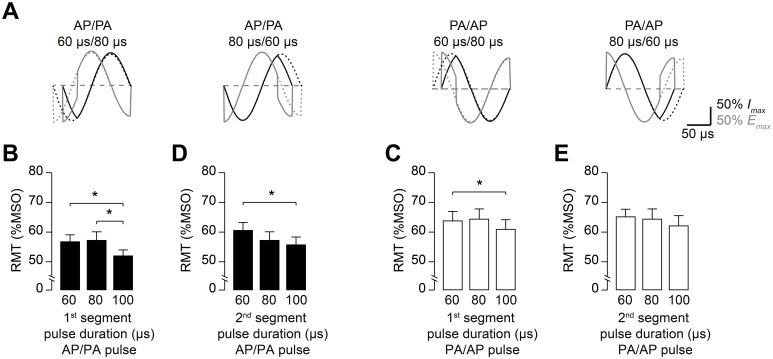
Changing half-segment duration of the full-sine pulse impacts on RMT. The duration of either the first or second half-segment of the full-sine pulse was modified for both current orientations from 80 µs to 60 or 100 µs, resulting in total pulse durations of 140 and 180 µs, respectively. (A) Examples of waveforms used for this experiment. Unaltered waveforms (duration of each half-segment 80 µs, total pulse duration 160 µs) are shown as dashed lines for comparison. (B) Length variation of the first half-segment of the AP/PA pulse. Prolonging the initial AP-oriented half-segment by 20 µs significantly decreased RMT. (C) Length variation of the second half-segment of the AP/PA pulse. (D) Length variation of the first segment of the PA/AP pulse. (E) Variation of the second segment of the PA/AP pulse did not result in significant RMT changes. Data are means (n = 10), error bars represent sem. *P<0.05, one-sample or paired *t* test on data normalized to the unaltered reference pulses.

(a) Pulse segment amplitude is referenced relative to the unaltered full-sine pulses (i.e. 100%/100% relative amplitude).

(b) RMT data were normalized to the unaltered reference pulses for statistical analysis because RMT was significantly different between AP-PA and PA-AP oriented full-sine pulses (cf. Figs. 5 and 6).

(c) Current direction refers to induced current in M1-HAND.

(d) P-values refer to results of post-hoc Student’s paired *t* tests between current orientations of the pulse (AP-PA vs. PA-AP).

(e) In experiment 3b, duration of a given half-segment was shortened or prolonged, resulting in total pulse durations of 140 or 180 µs, respectively.

We also varied the duration of the first or second half-segment of a given full-sine pulse in Experiment 3b, which had a similar design to experiment 3a (amplitude variation). The duration of either the first or second half-segment of the pulse was set to 60, 80 or 100 µs, respectively, whereas the other segment remained unaltered at 80 µs (Fig. 6A). Here, rmANOVA on normalized RMT data showed no pulse length * current direction×pulse segment interaction (*F*_(1;9)_ = 0.04, *P* = 0.85). There were main effects of pulse length (*F*_(1;9)_ = 35.0, *P*<0.001), of pulse segment (*F*_(1;9)_ = 19.5, *P* = 0.002), and a pulse length×current direction interaction (*F*_(1;9)_ = 7.9, *P* = 0.02). In general, RMT was lower for longer pulses regardless of the half-segment being modified (Fig. 6B–6E). Compared with the unaltered pulse, post-hoc tests were significant for one condition (prolongation of the first half-segment of the AP-PA pulse: *P* = 0.002, one sample *t* test; Fig. 6B).

**Figure 7:**
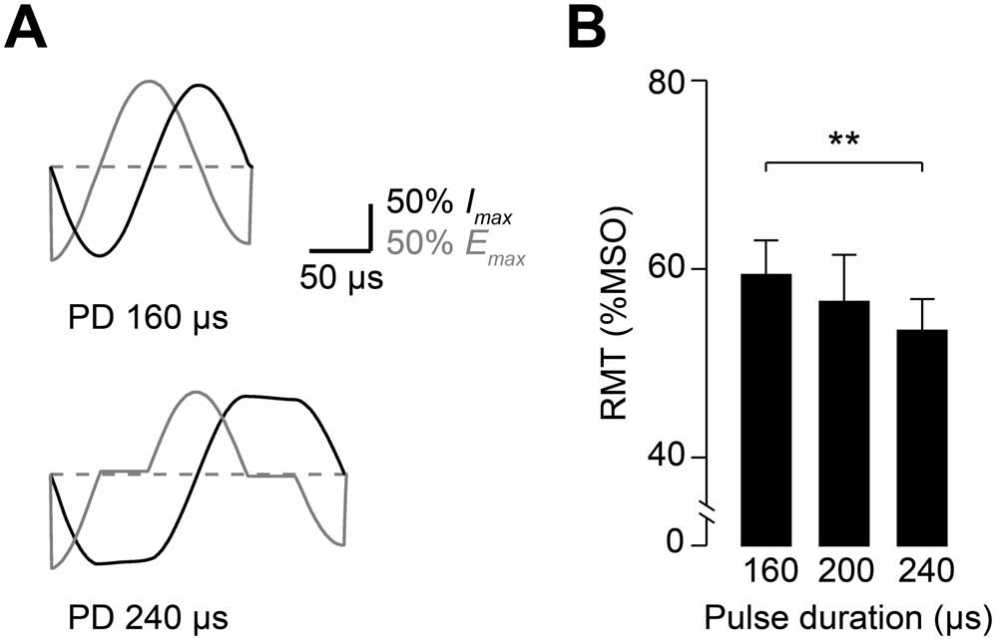
Longer full-sine pulses are characterized by lower RMT. Symmetrically prolonged full-sine (biphasic) waveforms impact on RMT. Pulses of 200 and 240 µs duration were compared with the unaltered 160 µs full-sine waveform. (A) Examples of waveforms used for Experiment 4. Note the middle phase of the electric field waveform *E* (gray lines) is identical for these pulses. The three phases of the induced electric field are temporally separated. (B) RMT probed with full-sine pulses of 160 µs, 200 µs, and 240 µs total duration. RMT was significantly lower for longer pulse duration. Data are means (n = 10), error bars represent sem. **P<0.01, Student’s paired *t* test.

In the experiments presented so far we modified the configuration of full-sine pulses to change the induced electric field waveform in its middle phase to a variable extent. This part of the waveform is assumed to be most relevant for stimulation [26], [27], [42]. To directly test if changes exclusively in other parts of the full-sine waveform also impact on RMT, we probed coil current waveforms that did not result in alterations of the middle part of the induced electric field. To this end, we modified full-sine pulses by adding periods with nearly zero electric field changes at the time of phase reversals (i.e. between the negative and positive electric field phases, Fig. 7A). Otherwise, the amplitude and shape of the electric field phases were not modified. We found that RMT decreased with increasing pulse duration (rmANOVA: *F*_(2;18)_ = 4.12, *P* = 0.034; Fig. 7B), and pulses of 240 µs duration had significantly lower RMT values than the unaltered 160 µs full-sine waveform (*P* = 0.005; Student’s paired *t* test). Thus, longer pulses were more effective although prolongation of the coil current waveform did not add largely to the induced electric field waveform. Since amplitude and shape of the three phases of the induced electric field were very similar for the waveforms tested in this experiment (Fig. 7A), we infer that the temporal separation of hyper- and depolarizing phases in the electric field waveform caused the decrease in RMT with longer pulse duration.

## Discussion

Using the same TMS device, we probed the impact of several modifications of the full-sine waveform shape on excitation of corticospinal output neurons in human M1-HAND. Our results show how order, current direction, and pulse duration of the two half-segments influence the stimulation effect of single-pulse TMS using the full-sine waveform.

### Cortical excitability probed with different pulse waveforms

Different resonance frequencies of stimulators and possible differences in coil design may influence and partly obscure the specific effect of TMS waveforms. In our study, distinct pulse shapes were applied through the same TMS device [12], enabling a direct comparison of waveform effects without confounding factors due to differences in stimulator design. Measurements of RMT, MEP amplitudes, and stimulus-response-curves with half-sine, full-sine, as well as monophasic TMS waveforms showed consistently that full-sine pulses had the highest effectiveness to elicit an MEP (Fig. 3). RMT is used in this context as measure of a waveform’s effectiveness to elicit a motor response [27]. Greater effectiveness of full-sine stimulation has previously been reported comparing full-sine and monophasic TMS pulses [13], [20]–[24], [40]. Using the RMT as indicator of corticospinal pathway excitation, our results confirm and extend these previous studies by showing superior effectiveness of full-sine relative to half-sine and monophasic TMS waveforms using a single stimulator to apply all three waveforms. Importantly, we can thus rule out that differences in stimulator design have a pronounced contribution to the distinct effectiveness of monophasic, half-sine and full-sine stimuli. The impact of full-sine TMS on RMT prompted further investigations into how the configuration of the full-sine waveform determines its stimulation effect.

### The shape of the full-sine waveform renders the TMS pulse more effective

When trying to assess which part of the full-sine waveform is responsible for stimulation effects or orientation-specific results, it has to be taken into account that the induced electric field *E*, which is determined by the rate of change of magnetic field over time [42], is most relevant for stimulation of neural tissue. Half-sine and full-sine TMS pulses induce an electric field that is more asymmetrical than that of the monophasic waveform (Fig. 2). This difference likely explains the stronger directional effects observed with monophasic TMS [13], [26]. When compared with the half-sine waveform, full-sine pulses are more effective and have similar directional effects with opposite initial current orientation. The full-sine pulse shape differs from half-sine in its duration and the induced electric field during the second half-segment of the waveform. The middle part of the full-sine waveform causes the longest change in magnetic flux having an opposite direction with respect to the first and third segment of the electric field waveform. This feature accounts for the direction-specific behavior of the unaltered full-sine pulse [13], [26].

Our findings provide direct experimental evidence that the shape of the waveform is responsible for the higher effectiveness of full-sine TMS pulses to elicit a motor response. Pulses with identical duration consisting of two concatenated half-sine segments had higher RMT than full-sine pulses and similar RMT to half-sine (Fig. 4). These results show that the shape of the full-sine waveform rather than its longer duration renders the full-sine waveform more effective than the half-sine waveform. The data also provide empirical evidence in support of the notion that the increased effectiveness in terms of M1-HAND stimulation is caused by the prolonged electric field phase in the middle part of the pulse [26], [27], [37], which is either positive or negative, depending on the initial current orientation of the full-sine TMS pulse.

### Which part of the full-sine waveform contributes most to the stimulation?

The two half-segments with opposite current orientation cause the prolonged electric field middle phase of the full-sine waveform. Modulations of any given half-segment therefore result in alterations of this middle phase and may reveal the relative contribution of the two distinct half-segments. Elucidating which half-segment of the waveform contributes most to the stimulation effect is essential for our understanding of the physiology of full-sine TMS. To address this question, previous studies explored direction specific effects of TMS using monophasic and full-sine (biphasic) waveforms [13], [26]. Direction specific effects of full-sine pulses resembled those of PA monophasic pulses, if full-sine pulses had AP-PA orientation, and were similar to AP monophasic stimulation when full-sine TMS was PA-AP oriented [13]. From these results the authors concluded that the second half-segment of the full-sine waveform might be most relevant for direction-specific effects [13]. Up to now, technical limitations have hampered direct experimental testing of this hypothesis, which motivated the variation of full-sine pulse half-segments in the present study. For AP-PA and PA-AP current orientations, changing the pulse half-segment resulting in PA current flow in M1-HAND had the largest impact on RMT (Fig. 5). In single-pulse TMS, PA current direction has stronger stimulation effects compared with AP current direction for monophasic and half-sine waveforms, which most likely results from recruitment of different target structures with lower threshold [13]–[15], [24], [43], [44]. Our results show that the half-segment of the full-sine pulse inducing PA current in M1-HAND contributes more strongly to neural stimulation, regardless of whether it is the first or second segment. Interestingly, the amplitude reduction in the first and second half-segment had very similar effect on the middle phase of the electric field waveform. However, RMT was consistently changed to a greater extent if the half-segment inducing PA current was altered (Fig. 5). This is surprising, since amplitude and duration of the middle phase are likely to be critical factors determining the effectiveness of full-sine pulses. Thus, our findings provide experimental evidence that excitation of neuronal target cells probably occurs in the half-segment of the full-sine pulse inducing PA oriented current [44]. Another possible explanation for our results might be that the distinct phases of the full-sine waveform activate different populations of cortical neurons. Indeed, PA and AP oriented stimuli are assumed to target different neuronal structures with different relative thresholds [10], [19], [43]. Because full-sine stimuli are composed of half-segments with PA and AP current orientation, different neuronal targets are likely to play a role for TMS using full-sine pulses.

The changes of pulse amplitude in our experiments altered the middle part of the induced electric field waveform more than changes in pulse length did. This difference may explain why alterations of pulse segment duration influenced RMT less than changes of pulse amplitude did (Fig. 6). Another implication of our findings is that the amplitude of the half-sine pulse segment causing AP oriented current in M1-HAND can be markedly reduced with unchanged effectiveness of TMS. Amplitude reduction might be important considering that coil heating scales non-linearly by the power of two with coil current amplitude [45].

### Implications for the stimulation effect of TMS

We also tested different pulses without altering the middle phase of the induced electric field waveform (Fig. 7). Although this part of the waveform remained unchanged, RMT decreased significantly with longer pulse duration. Thus, effectiveness of full-sine (biphasic) TMS is influenced by other parts of the waveform as well [13]. The additional electric field in the added waveform segments is nearly zero. Consequently, the negative and positive phases are separated by a short time interval. The two opposing segments of the full-sine pulse can be considered as primarily hyper- and depolarizing phases, and a sequence of hyperpolarization and subsequent depolarization was previously proposed as explanation for the higher effectiveness of full-sine stimulation [27]. The hyperpolarizing initial AP half-segment of a full-sine AP-PA pulse might lead to higher availability of sodium channels and thus enhance the depolarizing effect of the subsequent PA-oriented second half-segment [46]. Our results support this hypothesis by providing further experimental evidence. Alterations of the hyperpolarizing full-sine pulse half-segment inducing an AP oriented current had less effect than changes of the depolarizing PA half-segment (Fig. 5). Introducing a short delay between the negative and positive electric field phases reduced RMT (Fig. 7), which might act by enabling the hyperpolarization from the initial, negative electric field phase to subside more before the depolarizing (positive) middle phase. A recent modeling study demonstrated hyperpolarization effects by AP oriented parts of TMS waveforms for several different cortical neuron types [44]. The model could also reproduce the higher effectiveness of full-sine relative to monophasic pulses, and indicated that stimulation occurs through charge accumulation at axonal terminations [44]. Another possible explanation for the changes in RMT observed with the longer pulses in experiment 4 is that the hyper- and depolarizing segments of the waveform act as separated stimuli in short succession. Thus, the effectiveness of this pulse shape might also be due to temporal summation of excitatory postsynaptic potentials (EPSP) in M1-HAND.

The precise locus of excitation in M1-HAND and the specific neuronal target elements of TMS are still unresolved. Previous studies have addressed the locus of excitation using PET imaging [47] and modeling [48]. These data indicate that excitation and generation of early indirect waves (I-waves, [49]) likely occurs in cortical sulci [47] or their anterior wall [48]. Later I-waves might be generated at the crown of the precentral gyrus [48]. In our study, we compared the three commonly used TMS waveforms. The current orientation used for monophasic, half-sine, and full-sine pulses in our experiment 1 is thought to preferentially produce early I-waves [14], [39], probably originating from a similar site within M1-HAND [2], [48]. Accordingly, MEP latencies and stimulus-response curves for all three waveforms were comparable (Fig. 3E). The exact site of stimulation, however, is still not completely understood. And even within a certain cortical area, various types of neurons have different excitation thresholds for TMS [44]. It is thus conceivable that some of the observed threshold differences between waveforms are caused by recruitment of separate types of neurons in M1-HAND. In this context, our results elucidating the impact of different waveform parameters on the effectiveness to elicit a motor response may provide further important constraints for future modeling work.

### Limitations of the study

Due to technical reasons, the flexTMS stimulator generated switching transients as well as oscillations in coil current after the end of the pulse, which were picked up as high-frequency voltage by the search coil (Fig. 2). The high frequency (cycle period ∼15 µs) of these oscillations makes a physiological contribution to the stimulation effect unlikely, because neuronal membranes act as a low-pass filter with much longer time constant (∼150–200 µs, [50], [51]). These oscillations were present for all waveforms and should not bias the observed differences between waveforms. We can, however, not fully exclude higher-order effects on the stimulation.

For our investigation of novel TMS waveforms we focused on the effectiveness of single pulses to excite human M1-HAND as quantified by RMT. Since we did not study any other measures of cortical excitability with the different waveforms used in the present study, such as active motor threshold or cortical silent period, we cannot exclude differential effects of the tested TMS waveforms on these measures. Furthermore, our study does not allow any direct conclusions on how effectively TMS can induce plasticity, which may be strongly influenced by waveform characteristics and current orientation of the TMS pulse [21].

Another important parameter for TMS is spatial focality. Focality is mainly determined by the shape and design of the stimulation coil [52], [53], but the pulse waveform may also influence the focality of TMS-induced cortex activation. Focality of TMS, i.e. the surface area activated by a single pulse, is of particular importance for functional cortical mapping defining its spatial resolution [54]. In this context, a flexible TMS pulse design allowing modifications of a given segment of the TMS pulse might be an interesting option to achieve higher focality. Since we did not investigate if any of the novel waveforms were associated with increased focality, this issue needs to be addressed in future studies.

### Conclusion

In this study, we showed that the lower RMT of full-sine (biphasic) TMS is independent of stimulator design. Through flexible pulse design we were able to provide direct experimental evidence that the middle phase of the electric field waveform is critical for stimulation with full-sine pulses. Regarding the two oppositely directed half-segments of the full-sine waveform, we found that the relative contribution depends on current orientation of the pulse. The half-segment inducing a PA current is most important for the stimulation effect of full-sine pulses. Furthermore, RMT decreases with increasing duration of the full-sine waveform. Our findings contribute to a better understanding of the physiological effects of the biphasic full-sine waveform and might help improve future stimulator design for more efficient and precise non-invasive stimulation of the human brain.
